# Novel *Enterobacter* Lineage as Leading Cause of Nosocomial Outbreak Involving Carbapenemase-Producing Strains

**DOI:** 10.3201/eid2408.180151

**Published:** 2018-08

**Authors:** Racha Beyrouthy, Marion Barets, Elodie Marion, Cédric Dananché, Olivier Dauwalder, Frédéric Robin, Lauraine Gauthier, Agnès Jousset, Laurent Dortet, François Guérin, Thomas Bénet, Pierre Cassier, Philippe Vanhems, Richard Bonnet

**Affiliations:** Centre Hospitalier Universitaire, Clermont-Ferrand, France (R. Beyrouthy, F. Robin, R. Bonnet);; Centre National de Référence de la Résistance aux Antibiotiques, Clermont-Ferrand (R. Beyrouthy, F. Robin, R. Bonnet);; Université Clermont Auvergne, Clermont-Ferrand (R. Beyrouthy, F. Robin, R. Bonnet);; Institut National de la Santé et de la Recherche Médicale, Clermont-Ferrand (R. Beyrouthy, F. Robin, R. Bonnet);; Institut National de la Recherche Agronomique, Clermont-Ferrand (R. Beyrouthy, F. Robin, R. Bonnet);; Hôpital Édouard Herriot, Hospices Civils de Lyon, Lyon, France (M. Barets, E. Marion, C. Dananché, T. Bénet, P. Cassier, P. Vanhems);; Groupement Hospitalier Est, Hospices Civils de Lyon, Lyon (O. Dauwalder);; Centre International de Recherche en Infectiologie, Lyon (O. Dauwalder, T. Bénet, P. Vanhems),; Centre Hospitalier Universitaire Bicêtre, Paris, France (L. Gauthier, A. Jousset, L. Dortet);; Centre National de Référence Associé de la Résistance aux Antibiotiques, Paris (L. Gauthier, A. Jousset, L. Dortet);; Centre Hospitalier Universitaire, Caen, France (F. Guérin)

**Keywords:** Carbapenemase, Enterobacter cloacae complex, *Enterobacteriaceae*, beta-lactamase, nosocomial outbreak, VIM-4, next-generation sequencing, bacteria, antimicrobial resistance, carbapenemase-producing *Enterobacteriaceae*, CPE

## Abstract

We investigated unusual carbapenemase-producing *Enterobacter cloacae* complex isolates (n = 8) in the novel sequence type (ST) 873, which caused nosocomial infections in 2 hospitals in France. Whole-genome sequence typing showed the 1-year persistence of the epidemic strain, which harbored a *bla*_VIM-4_ ST1-IncHI2 plasmid, in 1 health institution and 2 closely related strains harboring *bla*_CTX-M-15_ in the other. These isolates formed a new subgroup in the *E*. *hormaechei* metacluster, according to their *hsp60* sequences and phylogenomic analysis. The average nucleotide identities, specific biochemical properties, and pangenomic and functional investigations of isolates suggested isolates of a novel species that had acquired genes associated with adhesion and mobility. The emergence of this novel *Enterobacter* phylogenetic lineage within hospitals should be closely monitored because of its ability to persist and spread.

Controlling the dissemination of carbapenemase-producing *Enterobacteriaceae* (CPE) is challenging because carbapenems are among the few antimicrobial drugs that can be used to treat severe infections in this family (*1*,*2*). Tzouvelekis et al. calculated the mortality rate of primary bacteremia involving CPEs without active therapy to be 54% (*3*). Thus, CPEs may carry the threat of a return to the pre–antimicrobial drug era.

The *Enterobacter cloacae* complex (ECC) has become the third most common species among CPEs in France (*4*). ECCs are not dominated by any single genotype (*5*), and only certain subspecies/species have previously been associated with infections and nosocomial outbreaks (*6*–*8*). The accurate identification of species and subspecies within the ECC is therefore needed to monitor outbreaks and infections.

The identification of species and subspecies within the ECC is challenging, and even more problematic because routine bacterial identification methods based on biochemical tests or matrix-assisted laser desorption/ionization time-of-flight mass spectrometry are yet unable to distinguish between them (*9*–*13*). In a seminal work, Hoffmann and Roggenkamp defined 13 genetic clusters (I –XIII) of the ECC on the basis of *hsp60* gene sequences and assigned them to species and subspecies (*14*). Recently, Chavda et al. extended the number of clusters in the ECC to 18 phylogenomic groups (A–R) by analyzing core single-nucleotide polymorphisms (SNPs) in 390 whole genomes (*15*).

Using whole-genome sequencing (WGS) approaches, we investigated a cluster of nosocomial carbapenemase-producing ECC isolates collected over a 13-month period in a university hospital in France. The results suggest a double-string diffusion mechanism involving the emergence of both a carbapenemase-encoding plasmid and an ECC phylogenetic lineage not previously described.

## Materials and Methods

### Clinical and Epidemiologic Survey

The patients were admitted to the Edouard Herriot Hospital in Lyon, France, where a prospective surveillance of CPEs has been implemented since 2012. Case-patients were defined as persons hospitalized after CPE was diagnosed in >1 clinical sample during January 12, 2014–December 31, 2015 (Technical Appendix Table 1). We investigated contact patients and performed an environmental study (Technical Appendix).

### Bacteria Isolation and Phenotypic Characterization

We isolated CPE on a chromogenic medium, chromID CARBA (bioMérieux, Marcy l’Etoile, France). We preincubated environmental CPE specimens for 24 h at 36°C in Trypticase soy broth (TSB; bioMérieux) supplemented with 0.5 mg/L ertapenem. The clinical (n = 7) and environmental (n = 2) isolates were identified by mass spectrometry (VitekMS; bioMérieux). We used the API 50CH system (bioMérieux) for biochemical testing and assessed antimicrobial susceptibility according to the EUCAST guidelines (http://www.eucast.org/). We detected carbapenemase by using the RAPIDEC CARBA-NP test (bioMérieux) (*16*). As previously described, we determined the sequence of *bla*_VIM-4_ by using the Sanger method (*17*). We performed conjugation experiments at 25°C as previously described (*18*) and plasmid size determination by pulsed-field gel electrophoresis (*19*). Biofilm formation and cell adhesion assays are described in the Technical Appendix.

### WGS and Genome Assembly

We determined the whole-genome sequences of strains C45 and C309 by using a hybrid de novo assembly of 2 × 150-bp paired-end reads generated by using sequencing technology by Illumina (San Diego, CA, USA) and long reads generated by using Pacific Biosciences technology (Menlo Park, CA, USA). We determined WGS of the other strains by using a de novo assembly of 2 × 150-bp paired-end reads. We performed the assemblies by using SPAdes (*20*), mapped the reads by using the Burrows-Wheeler aligner (BWA) (*21*), and polished the assembly by using Pilon (*22*). The raw data were deposited in EMBL as project PRJEB22398 and the assemblies as LT991954–60. We report further analysis processes in the online Technical Appendix.

## Results

### Emergence of Carbapenemase-Producing ECC strains

During January 12, 2014–December 31, 2015, a total of 320 positive cultures for ECC with antibiogram were identified in the Edouard Herriot Hospital in Lyon. Each of 7 (2.2%) ECCs recovered from 7 patients (designated P1–7) produced a carbapenemase (Table 1). These isolates were resistant to penicillins and combinations of penicillins, reacting by releasing β-lactamase inhibitors, oxyimino cephalosporins, and ertapenem (Table 2). PCR and sequencing showed the presence of the carbapenemase-encoding gene *bla*_VIM-4_ in the 7 isolates. During January 11, 2013–November 30, 2014, no positive cultures for ECC with antibiogram showed *bla*_VIM-4_. These results suggest an epidemic spread of VIM-4–producing ECC strains in the hospital.

**Table 1:** Case descriptions of VIM-4–producing *Enterobacter* *cloacae* complex nosocomial infections in outbreak involving carbapenemase-producing strains, Lyon, France, January 12, 2014–December 31, 2015*

Characteristic	Patient no.						
	P1	P2	P3	P4	P5	P6	P7
Patient age, y/sex	67/F	72/M	64/F	69/M	87/M	84/F	82/M
Hospitalization duration	10 d	106 d	52 d	26 d	6 d	69 d	36 d
Purpose of hospitalization	Kidney transplant	Peripheral arterial disease	Septic shock	Kidney transplant	Consciousness disorder	Necrotic purpura	Vesical lithotrity
Type of ward	Transplant, medicine	Surgery, medical, ICU	Medical, ICU	Transplant, medical	Medical, ICU	Medical	Surgery, medical, ICU
Antimicrobial therapy before diagnosis†	VAN, OFL, AMX	PTZ, VAN, AMI, MEM,† CTR	CTR, VAN, TZP, MET, AMI, CLI, IMP†	AMX, VAN	AMC	CTR, GEN	CTX, OFL, AMX, PTZ, GEN
Urinary catheter	Yes	Yes	Yes	Yes	Yes	No	Yes
Intubation	No	Yes	Yes	No	No	No	No
Central venous catheter	Yes	Yes	Yes	Yes	No	No	No
In-hospital death: delay from admission, d, and etiology	No	Yes: 106, septic shock from respiratory system	Yes: 52, septic shock, undetermined origin	No	No	No	Yes: 36, septic shock, peritonitis with hemorrhage
Last negative sample;‡ delay from admission, d	Urinary; 7	Urinary; 61	Urinary; 1	Urinary; 20	Rectal swab; 2	Necrotic skin; 39	Urinary; 2
First positive sample	Urinary	Urinary	Urinary	Urinary	Urinary	Necrotic Skin	Peritoneal fluid
Delay from admission, d	32	76	41	29	20	49	28
Delay from last negative sample, d	25	15	40	9	18	10	26
ST	ST873	ST873	ST118	ST873	ST118	ST873	ST110
Isolate identification	C45	C46	C47	C48	C308	C310	C309

*AMI, amikacin; AMX, amoxicillin; AMC, amoxicillin-clavulanate; CLI, clindamycin; CTR, ceftriaxone; CTX, cefotaxime; GEN, gentamicin; ICU, intensive care unit; ICU, intensive care unit; IMP, imipenem; MET, metronidazole; MEM, meropenem; OFL, ofloxacin; PTZ, piperacillin/tazobactam; ST, sequence type; VAN, vancomycin.  †Carbapenems. ‡For detection of VIM-4–producing *Enterobacter cloacae* complex.

**Table 2:** Key features of clinical VIM-4–producing *Enterobacter cloacae* complex isolates in nosocomial outbreak involving carbapenamase-producing strains, Lyon, France, January 12, 2014–December 31, 2015*

Isolate	Species	ST	hsp60 cluster (phylogenome)	Size of assembled genomes, bp	MICs, mg/L						
ETP	IPM	MEM	CAZ	CTX	FEP	ATM
C45	*E. cloacae* complex	873	NA (S)	5,290,194	2	2	1	32	>32	4	4
C46	*E. cloacae* complex	873	NA (S)	5,257,311	2	1	0.5	24	>32	2	4
C48	*E. cloacae* complex	873	NA (S)	5,260,873	2	2	0.5	24	>32	2	4
C310	*E. cloacae* complex	873	NA (S)	5,254,482	2	2	1	24	>32	2	2
E14	*E. cloacae* complex	873	NA (S)	5,251,662	2	2	1	32	>32	4	4
E16	*E. cloacae* complex	873	NA (S)	5,250,845	2	2	1	32	>32	4	4
C47	*E. cloacae* cluster III	118	III (D)	5,083,854	2	2	0.5	16	>32	2	0.25
C308	*E. cloacae* cluster III	118	III (D)	4,998,377	2	1	0.25	32	>32	2	0.5
C309	*E. hormaechei steigerwaltii*	110	VIII (B)	5,200,769	4	2	0.5	96	>32	8	32
*ATM, aztreonam; CAZ, ceftazidime; CTX, cefotaxime; ETP, ertapenem; FEP; IPM, imipenem; MEM, meropenem; NA, not applicable; ST, sequence type.											

### Temporal and Spatial Links of the Carbapenemase-Encoding ECC Cases

**Figure 1:**
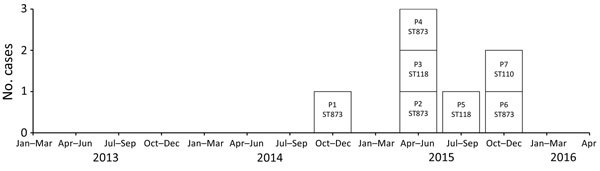
Epidemic curve of VIM-4–producing *Enterobacter cloacae* complex isolates (n = 7) in nosocomial outbreak involving carbapenamase-producing *Enterobacter* strains, Lyon, France, January 12, 2014–December 31, 2015. The attack rate was 0.7/10,000 hospital stays during the study period versus 0.0/10,000 hospital stays during January 11, 2013–November 30, 2014 (p = 0.008). The patients (P1–7) are labeled according to the ST of isolate with which they were infected or colonized. ST, sequence type.

The attack rate of CPE was 0.7/10,000 hospital stays during January 12, 2014–December 31, 2015, compared with 0.0/10,000 hospital stays during January 11, 2013–November 30, 2014 (p = 0.008; Figure 1). The all-causes crude mortality rate among patients with ECC isolates was 43% (n = 3). We provide additional clinical data in the online Technical Appendix. None of the patients had a recent history of travel or hospitalization in foreign countries. Only 2 patients (P6 and P7) were hospitalized in the same unit at the same time. Five patients (P1, P2, P3, P4, and P7) had undergone surgery in the same operating room but at different dates and with different operating teams. We identified CPE in urine samples of 5 patients (P1–P5) that were drawn from the patients’ urinary catheters. CPE was isolated from a skin sample that we excised from a necrotic ulcer from P6 and in a sample of operative peritoneal fluid from P7. Patient P1 was simply colonized, whereas patients P2–P7 were infected. All patients had >1 negative samples from the same clinical site during hospitalization before colonization/infection by ECC (Table 1). These findings showed no clear-cut epidemiologic or temporal links between the VIM-4 ECC case-patients, except that surgical procedures were performed in the same operating room on 5 of the 7 patients.

### Environmental Investigations

Putative sources previously described in other settings, such as handwashing sinks (*23*) and endoscopes (*24*), were not assumed to be a source of *bla*_VIM-4_ ECC because surveillance samples were negative during the study period. In addition to the 102 contact patients, we screened 65 persons during the first 5 episodes (illnesses in P1–P5) without identifying any secondary cases. For the last episode, which involved P6 and P7, we screened 125 of 160 contact patients; all were negative. These findings support a key role for 1 or multiple environmental reservoirs in the nosocomial transmission of *bla*_VIM-4_ ECC to patients. However, transmission by healthcare workers (HCWs) cannot be completely ruled out. We did not screen HCWs for possible CPE carriage, but no particular HCW was involved in care of all CPE cases. 

The data, including the temporal distribution of the cases over 13 months and the molecular characterization of the isolates, suggested intermittent transmission of human or environmental origin. We therefore implemented environmental screening in 3 rooms occupied by patients P3 and P7, including the beds, mattress covers, and shared equipment, in June and August 2016. These rooms were investigated because they had been occupied >1 time by patients with VIM-4–producing ECC (data not shown). Analysis of the environmental samples after the discharge of patients with VIM-4–producing ECC showed that those collected from a radiator and the mattress cover in 1 patient’s room (P7) were contaminated by VIM-4–producing ECC isolates (E14 and E16). The antibedsore mattresses were used in different rooms for several patients. The incidence of CPE was reduced by discarding the mattress covers after the patients known to be VIM-4–producing ECC carriers were discharged from the hospital (data not shown). These data suggest that the transmission of ECCs by contact with mattress covers could be 1 of the key causative factors, especially for the last episode (P6–P7).

### Multiclonal Spread of the VIM-4 Carbapenemase

**Figure 2:**
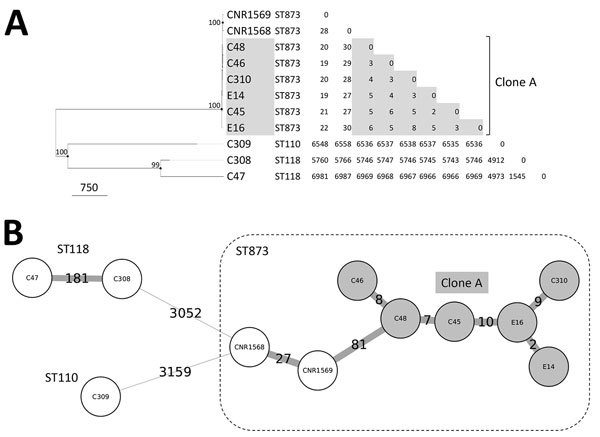
Whole-genome typing of *Enterobacter cloacae* complex isolates from nosocomial outbreak involving carbapenamase-producing *Enterobacter* strains, Lyon, France, January 12, 2014–December 31, 2015. A) Dendrogram inferred by the maximum-likelihood method on the basis of core genome SNPs. The node sizes are proportional to the bootstrap values; values >80 are indicated. Scale bar indicates SNPs. The relatedness of the strains was determined by using <15 variant sites as clonality criteria. B) Minimum-spanning tree based on a whole-genome multilocus sequence typing approach, combining the analysis of core genome loci and the presence or absence of accessory genes. Labels on branches indicate the absolute number of variant loci (clonality threshold <10 variant loci). SNP, single-nucleotide polymorphism; ST, sequence type.

We sequenced the genomes of isolates to assess the molecular links between the VIM-4 ECC cases (Table 2). Six isolates (C45, C46, C48, C310, E14, and E16) formed a clonal cluster designated clone A, in which core genome SNP analysis showed a diversion of <10 SNPs and wgMLST. (Figure 2).

**Figure 3:**
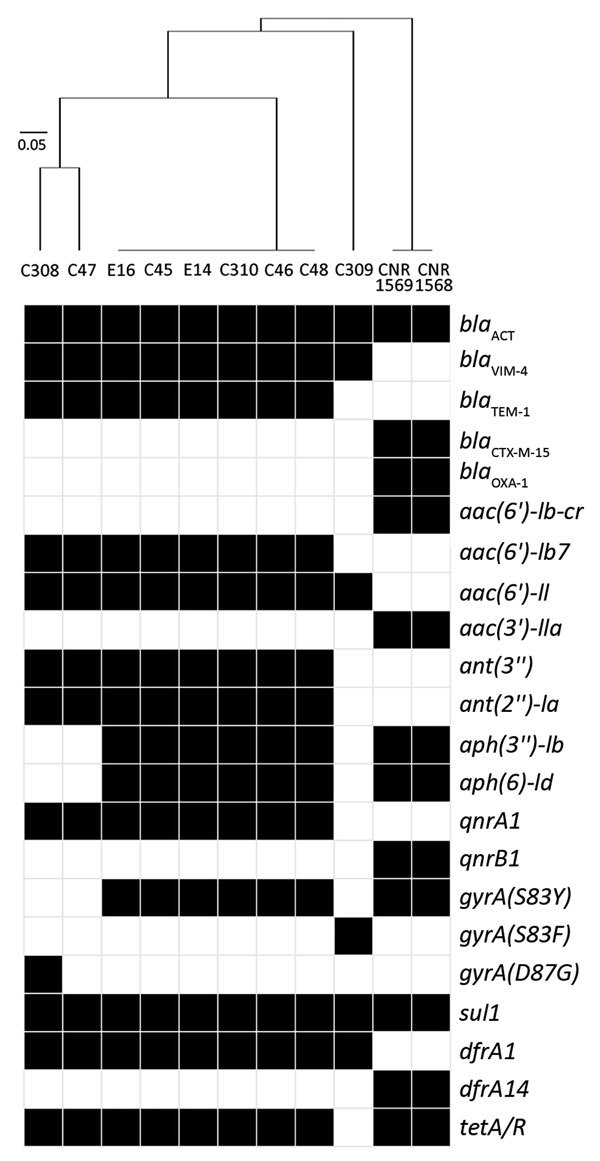
Genetic resistance determinants in *Enterobacter cloacae* complex isolates from nosocomial outbreak involving carbapenamase-producing *Enterobacter* strains, Lyon, France, January 12, 2014–December 31, 2015. Black cells indicate presence and white cells absence of resistance determinants. The isolates were classified according to the content in resistance determinants by using a binary distance matrix and UPGMA clustering method. Scale bar indicates the dissimilarity in resistance gene content.

Isolate C309 belonged to ST110, isolates C47 and C308 to ST118, and the 6 isolates of clone A to ST873 (*dnaA*:85/*fusA*:63/*gyrB*:101/*leuS*:103/*pyrG*:96/*rplB*:6/*rpoB*:53). By screening a collection of 30 ECCs isolated in France during the same period, we identified 2 ST873 isolates (CNR1568 and CNR1569) containing the extended-spectrum β-lactamase–encoding gene *bla*_CTX-M-15_ in the teaching hospital of Caen. These related isolates differed by 28 core genome SNPs and 27 loci (Figure 2). The clustering of the ECC isolates was also apparent in the analysis of the antimicrobial resistance gene contents (Figure 3). Overall, these data show a multiclonal spread of *bla*_VIM-4_ ECCs, the predominance of ECC genotype ST873 among *bla*_VIM-4_ ECC, and the interregional spread of this ECC lineage.

### VIM-4–Encoding Plasmids

To explore possible links between clone A and the other VIM-4–encoding isolates, we investigated the plasmid contents and the transferability of *bla*_VIM-4_ by conjugation. The transfer of ertapenem resistance into *Escherichia coli* C600 was successful; all of the 9 isolates were at room temperature. Hybridization of plasmids with a specific probe revealed the location of *bla*_VIM-4_ on conjugative plasmids of ≈300 kb (n = 6) in the clone A isolates, C47, and C308 and of ≈245 kb (n = 1) in isolate C309. In all isolates, analysis of the assembled genomes showed the presence of an ST1-IncHI2 replicon encoding *bla*_VIM-4_.

**Figure 4:**
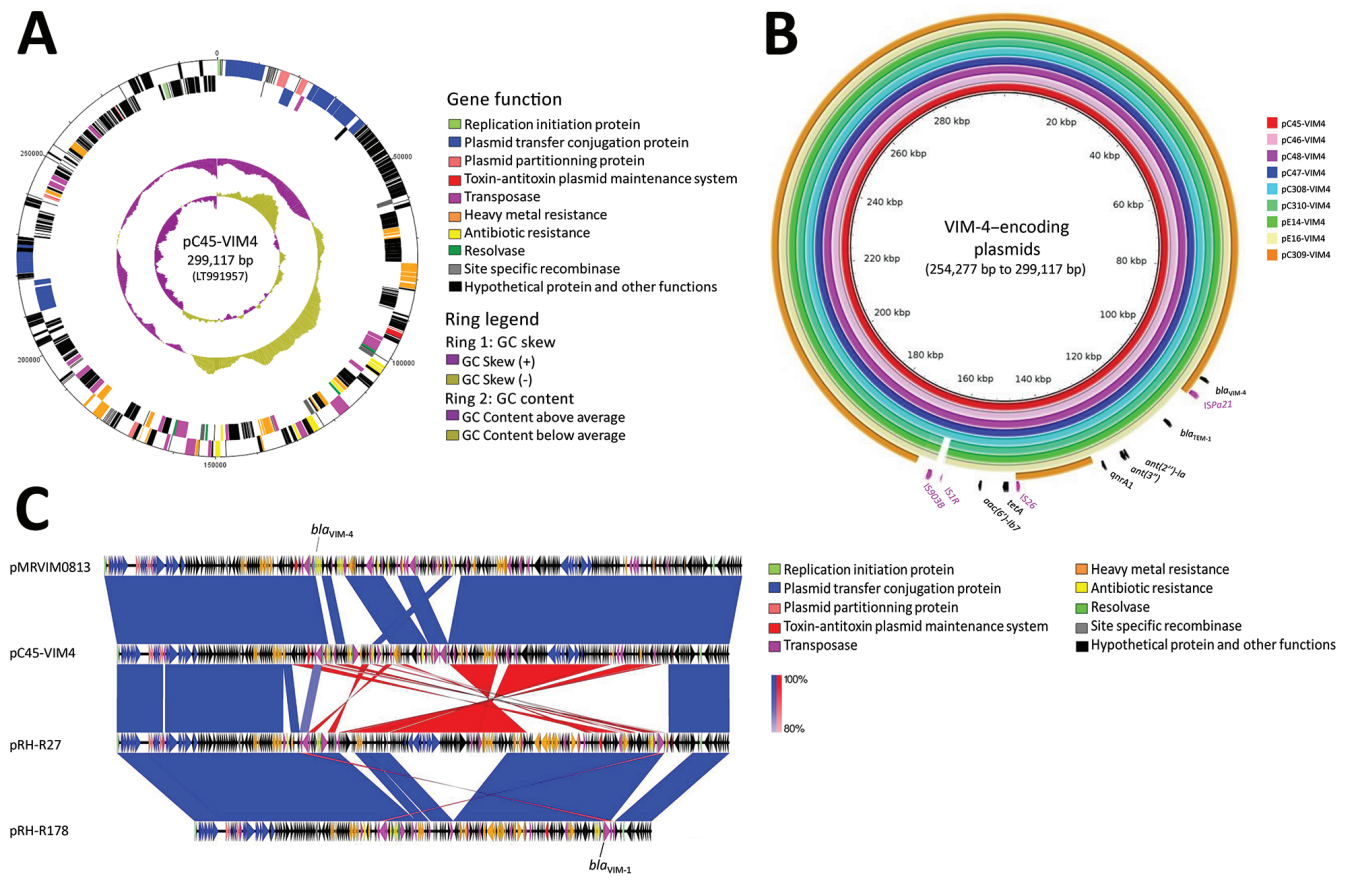
Analysis of *bla*_VIM-4_–encoding plasmids from study of nosocomial outbreak involving carbapenamase-producing *Enterobacter* strains, Lyon, France, January 12, 2014–December 31, 2015. A) Schematic representation of ST1-IncHI2 plasmid pC45-VIM4. The first ring indicates the coordinates of the complete plasmid circle. The 2 outer rings represent the forward and reverse open reading frames, respectively. B) Comparative sequence analysis of ST1-IncHI2 *bla*_VIM-4_–encoding plasmids from this study. The plasmids of isolates C45, C46, C47, C48, C308, C309, C310, E14, and E16 are designated pC45-VIM4, pC46-VIM4, pC47-VIM4, pC48-VIM4, pC308-VIM4, pC310-VIM4, pE14-VIM4, pE16-VIM4, and pC309-VIM4, respectively. C) Comparative sequence analysis of *bla*_VIM-4_–encoding plasmid pC45-VIM4 to the related *bla*_VIM-1_–encoding IncHI2 plasmids pMRVIM0813 (GenBank accession no. KP975077), pRH-R27 (GenBank accession no. LN555650), and pRH-R178 (GenBank accession no. HG530658). Vertical blocks between sequences indicate regions of shared similarity shaded according to blastn (https://blast.ncbi.nlm.nih.gov/Blast.cgi). Blue indicates matches in the same direction; red indicates inverted matches.

The plasmid from isolate C45 designated pC45-VIM4 formed a circular 299,117-bp sequence (Figure 4, panel A). The antimicrobial drug resistance genes were located in a ≈50-kb region (bases 97,253–154,784); *bla*_VIM-4_ gene was included in a 16kb Tn*21*-like transposon designated Tn*6540* (bases 97,253–113,368). Tn*6540* comprised a class 1 integron including *bla*_VIM-4_ as the first gene cassette, followed by *aac*(*6′*)*-Il*, *dfrA1b*, *Δant*(*3′′*)*,* and *smr2*. Seven heavy metal resistance loci were also encoded by pC45-VIM4: the tellurite resistance genes *terY3Y2XY1W* (bases 65,568–69,439) and *terZABCDEF* (bases 76,028–82,281); the cobalt-zinc-cadmium-resistance protein (bases 118,576 to 119,544); operon *copS*/*copE* (bases 165,340 to 167,337); the efflux system *rcnR*/*rcnA* (bases 167,595 to 169,105); the arsenic resistance genes *arsCBRH* (bases 181,666–184,550 bp); and 1 complete mercury resistance operon, *merRTCADE* (base 134,519–138,533).

We identified a similar organization in the other *bla*_VIM-4_ isolates (Figure 4, panel B). However, in C309, the VIM-4–encoding plasmid designated pC309-VIM4 (254,277-bp) differed by 2 deletion sites (≈24 kb and ≈21 kb) flanked by mobile elements (IS*26* and IS*Pa21*). These deletions resulted in the loss of resistance genes *aac*(*6′*)*-Ib7*, *tetA*, *bla*_TEM-1_, *ant*(*3′′*), and *ant*(*2′′*)*-*Ia. These results suggest the horizontal transfer of the same *bla*_VIM-4_–encoding plasmid in several lineages of ECC.

Three related IncHI2 plasmids encoding *bla*_VIM-1_ were identified in GenBank (Figure 4, panel C). Except in the region encoding resistance genes, pC45-VIM4 shared 94% of its sequence and most of gene synteny with pMRVIM0813; pRH-R27 and pRH-R178 reported from Germany are more distantly related (85% and 60% of overlap).

### A New Cluster in the Phylogenomic Tree of the ECC

Because specific subgroups within the ECC are more prone to cause nosocomial infections or outbreaks, we characterized the isolates at the species and subspecies levels as described by Hoffmann and Roggenkamp (*14*) and Chavda et al. (*15*). In the *hsp60*-based neighbor-joining tree comprising 52 representative reference and type strains (Technical Appendix Figure 1), the sequences of C47 and C308 co-localized with *hsp*60 cluster III and that of C309 localized with *hsp*60 cluster VIII; both had >99.3% identity within the clusters. The *hsp60* sequences of the ST873 isolates formed a new cluster that shared only 96.7%–97.1% identity with the closest related sequences.

**Figure 5:**
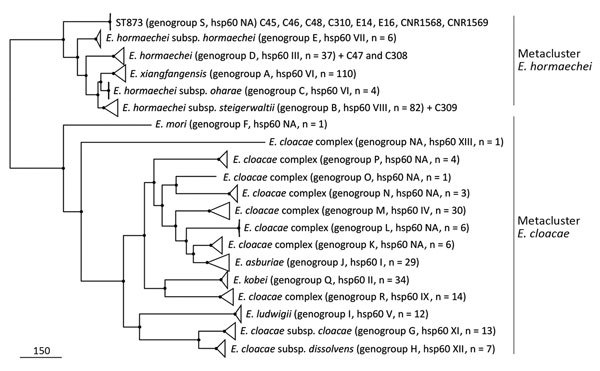
Approximately maximum-likelihood phylogenetic trees based on recombination free core single-nucleotide polymorphisms (SNPs) inferred from ST873, ST110 and ST118 genomes and 398 representative genomes of *Enterobacter cloacae* complex strains in study of nosocomial outbreak involving carbapenamase-producing *Enterobacter* strains, Lyon, France, January 12, 2014–December 31, 2015. All nodes are supported by Shimodaira-Hasegawa test values >97%. Scale bar indicates SNPs. NA, nonattributed; ST, sequence type.

To confirm that the ST873 isolates formed a new subgroup, we performed a phylogenomic analysis with 398 ECC genomes downloaded from GenBank. In the resulting phylogenomic tree (Figure 5), the genomes were distributed in 2 major branches corresponding to the *E. hormaechei* and *E. cloacae* metaclusters as previously reported (*14*,*15*). The *E. hormaechei* metacluster comprised 6 branches corresponding to Chavda’s phylogenomic groups A–E, and a new phylogenomic group, designated S, comprising solely the ST873 isolates. As expected, the C47, C308, and C309 isolates clustered in Chavda’s phylogenomic groups D and B, which correspond to Hoffmann’s *hsp60* clusters III and VIII (Figure 5). These findings fortify the hypothesis that the ST873 isolates could be a new species or subspecies in the *E. hormaechei* metacluster.

### A New Species in the *E.* *hormaechei* Metacluster

**Figure 6:**
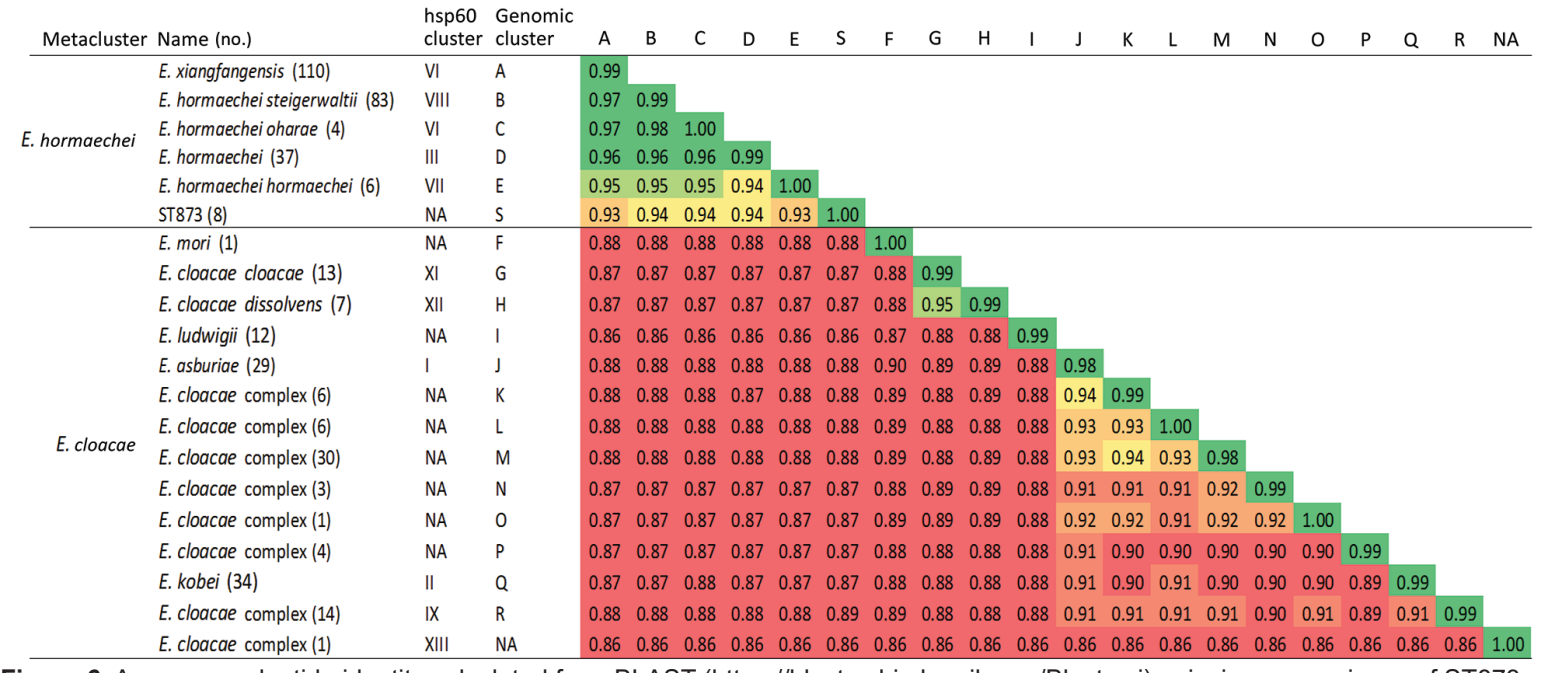
Average nucleotide identity calculated from BLAST (https://blast.ncbi.nlm.nih.gov/Blast.cgi) pairwise comparisons of ST873 genomes and 398 *Enterobacter cloacae* complex genomes in study of nosocomial outbreak involving carbapenamase-producing *Enterobacter* strains, Lyon, France, January 12, 2014–December 31, 2015. NA, nonattributed.

Average nucleotide identity (ANI) and percentage of conserved DNA (PCD) can accurately replace DNA–DNA hybridization values for species delineation by using 0.95 and 0.69 as ANI and PCD thresholds, respectively (*25*,*26*). We therefore calculated ANIs and PCDs for the ST873 isolates against 398 ECC genomes by using BLAST (http://blast.ncbi.nlm.nih.gov/Blast.cgi). The PCD values were high enough (>0.69) within the *E. hormaechei* and *E. cloacae* metaclusters for the delineation of species and subspecies by ANI calculations (Technical Appendix Figure 2). Genomes within the same phylogenomic group shared ANI mean values >98% (Figure 6). In the *E. cloacae* metacluster, the ANI values supported the designation of the phylogenomic groups as different species (ANI, 0.87–0.94), except for *E. cloacae* subsp. *cloacae* and *E. cloacae* subsp. *dissolvens* (ANI, 0.95). In the *E. hormaechei* group, most phylogenomic groups shared ANI values 0.96–0.98, supporting the split of the *E. hormaechei* metacluster into different subspecies. As expected, isolates C47 and C308 clustered in the *E. hormaechei* phylogenomic group D and isolate C309 in the phylogenomic group B (also designated *E. hormaechei* subsp. *steigerwaltii*). The new phylogenomic group S, comprising the ST873 isolates, had ANI values below the species cutoff (<0.95) against all groups, including those in the *E. hormaechei* metacluster. We obtained similar results by using the MUMmer-based approach (Technical Appendix Figures 3, 4), suggesting that the ST-873 isolates are a new species of the *E. hormaechei* metacluster.

The 3 subspecies *E. hormaechei* subsp. *hormaechei*, *E. hormaechei* subsp. *oharae*, and *E. hormaechei* subsp. *steigerwaltii* can be differentiated by using D-adonitol, D-arabitol, D-sorbitol, and D-melibiose fermentation tests (bioMérieux). By using the API 50CH system, we found that the biochemical characterization of our isolates yielded results compatible with *E. hormaechei* subsp. *steigerwaltii* (*hsp60* cluster VIII and phylogenomic group B), as expected (Technical Appendix Tables 1, 2). Of interest, the ST873 isolates produced a distinguishable biochemical phenotype in the *E.* *hormaechei* metacluster by growing on only D-melibiose as the sole carbon source (Technical Appendix Table 2). Overall, our molecular and biochemical data agree with those of previous studies in the field and suggest that the ST873 isolates are a new species of the *E.* *hormaechei* metacluster.

### Functional Genomics in the *E. hormaechei* Metacluster

**Figure 7:**
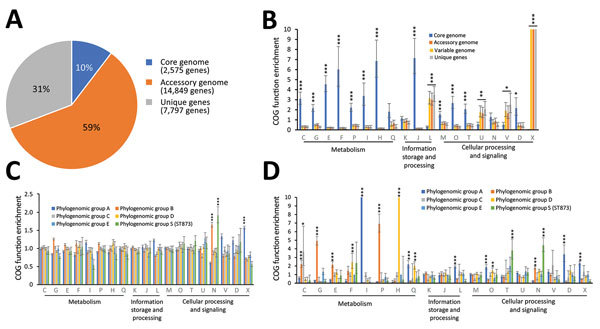
Pangenome analysis of metacluster *Enterobacter hormaechei* in study of nosocomial outbreak involving carbapenamase-producing *Enterobacter* strains, Lyon, France, January 12, 2014–December 31, 2015. A) Distribution of COGs); B) functional annotations in the pangenome; C) functional annotations in the variable genome (accessory genome + unique genes); and D) functional annotations for specific genes. Bar charts show the enrichment of COG categories as odds ratios; error bars indicate 95% CIs. Asterisks indicate certain COG categories that are significantly enriched: *p<0.05; **p<0.01;***p<0.001, all by Fisher exact test. Each COG category is identified by a 1-letter abbreviation: C, energy production and conversion; D, cell cycle control and mitosis; E, amino acid metabolism and transport; F, nucleotide metabolism and transport; G, carbohydrate metabolism and transport; H, coenzyme metabolism; I, lipid metabolism; J, translation; K, transcription; L, replication, recombination and repair; M, cell wall/membrane/envelope biogenesis; N, cell motility; O, post-translational modification, protein turnover, and chaperone functions; P, inorganic ion transport and metabolism; Q, secondary metabolism; T, signal transduction; U, intracellular trafficking and secretion; V, defense mechanisms; and X, mobilome. COG, clusters of orthologous groups.

To investigate the functional features of the ST873 isolates and other phylogenomic subgroups in the *E.* *hormaechei* metacluster, we constructed a pangenome by using 245 strains including our isolates. The pangenome was divided into 3 sections: 1) the core genome (the set of genes shared by 99% of strains), 2) the accessory genome (the set of genes present in some but not all representatives), and 3) the unique genome (genes unique to individual strains). The 245 strains examined yielded a pangenome of 25,221 genes. On the basis of this dataset, the core genome is composed of 2,575 genes, the accessory genome of 14,849 genes, and the unique genome of 7,797 genes (Figure 7, panel A). Functional annotation of the pangenome on the COG database showed an overrepresentation of genes belonging to the groups with housekeeping functions (COG categories C, G, E, F, I, P, H, J, O, D, and T) in the core genome (Figure 7, panel B). The accessory genome and the unique genes had a similar distribution of functional annotations with an overrepresentation of genes involved in DNA recombination (genes encoding integrases, transposases, or resolvases) and defense mechanisms, as well as those belonging to the mobilome (COG categories L, U, V, and X), as expected.

The distribution of variable genome annotations among the phylogenomic groups showed closely related distributions of functional annotations (Figure 7, panel C), except for cell mobility annotations (COG category N, comprising the flagellar- and adhesion-related functions), which were overrepresented in phylogenomic group B and the ST873 isolates (phylogenomic group S), and mobilome annotations (COG category X), which were overrepresented in phylogenomic group A. The differences in functional distribution were enhanced by the analysis of variable genes specific to phylogenomic groups (Figure 7, panel D). Phylogenomic group B (*hsp60* group VIII), the most prevalent subgroup in human infections, and the new epidemic phylogenomic group S (ST873) had an overrepresentation of genes involved in cell motility (COG category N), as previously observed among variable genes, showing that these functions are overrepresented and based on specific genes in these ECC lineages. The ST873 group also exhibited enrichment in specific genes related to signal transduction (COG category T), and phylogenomic group A exhibited enrichment in genes related to recombination and the mobilome (COG categories L and X). We also observed other differences in genes related to metabolic functions. Phylogenomic group A demonstrated an enrichment in specific genes involved in lipid metabolism (COG category I), and phylogenomic group D has specific genes involved in coenzyme, nucleotide, and secondary metabolism (COG categories F, H and Q); phylogenomic group B accumulated specific genes involved in energy production and carbohydrate, amino acid, and ion metabolism (COG categories C, G, E and P). Overall, these data suggest that there exist distinct lifestyles in ECCs explaining varied abilities to colonize the hospital environment and to induce nosocomial infections.

### Adhesion to Abiotic Surface and Epithelial Cells

To investigate the overrepresentation of mobility/adhesion functions in the ST873 isolates in the context of their prolonged persistence in hospital, we compared the ability of our isolates to initiate biofilm formation on an abiotic surface and their adhesion to intestinal epithelial cells. The ST873 isolates had greater ability to initiate biofilm on PVC than did isolates of ST118 and ST110 (Technical Appendix Figure 5, panel A). We also observed slight differences of adhesion to HT29 intestinal epithelial cells (Technical Appendix Figure 5, panel B). These results suggest that ST873 isolates have original adhesion features, as suggested by the pangenomic analysis.

## Discussion

One major issue regarding CPEs is whether the main driver of the spread of carbapenemases is the transmission of successful clonal lineages or the horizontal transfer of carbapenemase genes by mobile genetic elements such as plasmids. Our study provides evidence for the spread of an epidemic VIM-4–encoding IncHI2 plasmid in distinct lineages of the ECC and the 1-year persistence of an epidemic strain ST-873, suggesting a double-string diffusion mechanism involving the emergence of both a VIM-4–encoding plasmid and a persistent ECC phylogenetic lineage.

ECC accounted for 9.4% and VIM-4–producing ECC for 3.9% of CPEs during 2015 in France (*27*). Only 1 additional case of a VIM-4–producing ECC was observed in Paris during the same period, and only 16 VIM-4–producing *Enterobacteriaceae* cases were diagnosed: the 7 ECC cases included in this study, 6 cases of *E. coli* in the Paris area, and 2 cases of *Citrobacter freundii* in the north of France. During this period, 6 other cases of CPE were identified in the same hospital: 5 produced oxacillinase 48 carbapenemases, and 1 produced New Delhi metallo-β-lactamase 1; these cases, in line with common observations in France and other countries in Europe, were imported (*4*). Our investigation was therefore prompted by the contrast between the low incidence of VIM-4–producing ECC cases in France and our case series, which suggested a new carbapenemase-spreading factor.

All the VIM-4 isolates we found harbored a similar *bla*_VIM-4_–encoding, self-conjugative plasmid. The *bla*_VIM-4_ gene has previously been reported only in IncA/C plasmids in a neighboring country (Italy) (*28*). The epidemic plasmid we identified belongs to the ST1-IncHI2 incompatibility group. IncHI2 plasmids are frequent among the ECCs and are often associated with the dissemination of genes encoding extended-spectrum β-lactamases and, at least to some extent, *bla*_VIM-1_ (*29*). Although *bla*_VIM-4_ differs from *bla*_VIM-1_ by a point mutation, the IncHI2 *bla*_VIM-4_ plasmids we identified substantially diverge from previously reported *bla*_VIM-1_ plasmids by the ≈50kb region encoding the associated resistance genes.

The VIM-4 epidemic strain ST873 persisted in the hospital for ≈1 year despite the application of specific isolation precautions for patients colonized or infected. During the investigation of another nosocomial outbreak that occurred in 2016 in Caen University Hospital (692 km from Lyon), we identified 2 CTX-M-15–encoding ECC strains belonging to ST873, which suggests that the spread of ST873 is not geographically limited. These strains had traits of speciation and specific genes related to signal transduction, cell motility, and adhesion. These functions have a crucial role in the initiation of biofilm formation (*30*), which was enhanced in the ST873 isolates compared with the other VIM-4 isolates in our study. Biofilm formation, a key function for host–pathogen interactions and environmental survival, may explain the successful persistence of ST873 isolates in the hospital settings of this study.

Nosocomial infections mediated by *Enterobacteriaceae* can be transmitted to patients in medical settings by HCWs, patient-to-patient spread, or environmental sources (*31*). Our findings emphasize the need to consider clinical circumstances such as bed contamination, as previously observed (*32*). In addition, the VIM-4֪–encoding plasmid pECC-VIM4 transferred to a bacterial recipient at room temperature, which could explain why successful horizontal transfer into ECC multiclonal isolates occurred in the hospital environment and provided support for the crucial importance of environmental reservoirs in the transmission of nosocomial pathogens. Hence, the threat of outbreaks can be limited by high-quality cleaning and disinfection of patient-care areas and the regular replacement of equipment such as mattress covers.

In conclusion, we report a nosocomial outbreak of multiclonal VIM-4–producing ECC that originated from contamination in the hospital environment. The predominant clone belongs to a new lineage in the ECC and should be closely monitored in the context of nosocomial infections caused by its apparent ability to maintain and spread in a hospital setting. Our study also delineated the multifactorial spread of the VIM-4 carbapenemase and emphasizes the usefulness of ECC phylogenomic typing in the investigation of outbreaks.

Technical Appendix Additional information on study of nosocomial outbreak involving carbapenamase-producing *Enterobacter* strains, Lyon, France, January 12, 2014–December 31, 2015.
