# Supplementary material for: Novel Enterobacter Lineage as Leading Cause of Nosocomial Outbreak Involving Carbapenemase-Producing Strains
Source: Emerg Infect Dis. 2018 Aug;24(8):1505–15. doi: 10.3201/eid2408.180151 (PMC6056098; doi:10.3201/eid2408.180151)
Supplement: Technical Appendix — Additional information on study of nosocomial outbreak involving carbapenamase-producing Enterobacter strains, Lyon, France, January 12, 2014–December 31, 2015. [file 18-0151-Techapp-s1.pdf]

Article DOI: https://doi.org/10.3201/eid2407.171313

Novel *Enterobacter* Lineage as Leading Cause of Nosocomial Outbreak Involving Carbapenemase-Producing Strains

Technical Appendix

Material and Methods

Contact Patients and Environment Study

In accordance with French recommendations (http://www.hcsp.fr), contact patients were defined as patients hospitalized in the same unit as a carbapenemase-producing *Enterobacteriaceae* (CPE) carrier and/or managed by the same medical or nursing staff. These patients were screened weekly for CPE by rectal swab. All CPE carriers were placed on strict contact precautions with dedicated staff as soon as they were identified. In addition, three rooms for CPE patients including the beds, mattress covers, and shared equipment were sampled after cleaning, either with swabs (Copan, Brescia, Italy) or with sterile wipes (bioMérieux, Marcy l'Etoile, France). Each epidemiologic investigation included on-site visits and the collection of patient demographic characteristics, hospitalization details, and clinical and bacteriological data. The attack rate over the 13-month period was calculated for 10,000 hospital stays and compared with that of the previous 13-month period (11/01/2013 to 11/31/2014) using Fisher exact test. $p < 0.05$ was considered significant.

wgMLST and Core Genome SNP-based Typing

The wgMLST approach combined the analysis of core genome loci and the presence/absence of accessory genes using BWA read alignments as previously described (*1*). Core and accessory genes were extracted from 60 genomes belonging to the hormaechei metacluster, yielding 2315 core genome loci (2,115,786 bases) and 2091 accessory genes (2,109,600 bases). SNP-based typing was based on the best practices guide produced by the Broad Institute (*2*). After quality trimming with sickle, the reads were aligned against the core

genome with BWA-mem. PCR duplicate removal and read realignment around indels were then performed with Picard tools (http://broadinstitute.github.io/picard/). Following this alignment processing, variant calling and variant filtering were performed with VarScan (*3*). As recombination events mask the true phylogenetic signal by generating tight clusters of SNPs, SNPs were filtered out in each mapping if they were called within the vicinity of 25 bp of another SNP, as previously performed (*4*). Core genomes with all variants instantiated were then generated from filtered SNPs to obtain the phylogenetic tree using RAxML (*5*) with 100 bootstrap replicates.

Gene Detection from NGS Data

Multilocus sequence typing (MLST) and plasmid double locus sequence typing (pDLST) were performed from online resources (http://pubmlst.org) with the ARIBA package (*6*). Replicons were detected by PlasmidFinder (http://www.genomicepidemiology.org/). The antibiotic resistance genes were assembled with the ARIBA package by mapping short reads against a manually curated and updated database of resistance-associated genes (2835 genes and 1308 SNPs from 130 genes) derived from CARD and Resfinder (*7,8*). The assembled genes were filtered and identified by alignment against the database with Exonerate (*9*) using a 95% minimum threshold for coverage and identity percentages. The distance presence/absence matrix derived from resistance gene detection and the corresponding UPGMA tree were computed in R (https://cran.r-project.org/) with ade4 (https://cran.r-project.org/web/packages/ade4) and fastcluster (https://cran.r-project.org/web/packages/fastcluster) packages.

SNP and hsp60 *Enterobacter cloacae* Complex Trees

The 85,257 SNPs were identified by Parsnp (*10*) using the recombination filtering option. These SNPs were used to infer an approximately-maximum-likelihood tree with FastTree (*11*). The reliability of the nodes was assessed by the Shimodaira-Hasegawa test. The phylogenetic tree was visualized with FigTree (http://tree.bio.ed.ac.uk/software/figtree). hsp60 typing was performed as previously described (*12*).

ANI and PCD Calculation

The average nucleotide identity (ANI) and the percentage of conserved DNA (PCD) were calculated as previously described (*13*) with the pyani module (https://github.com/widdowquinn/pyani).

Pangenome Construction

To determine the pangenome of the hormaechei metacluster, 244 genomes were annotated by Prokka (*14*) and the corresponding dataset was analyzed with the Roary package (*15*). The homologous genes were functionally annotated with the Clusters of Orthologous Groups (COG) database (*16*) using EggNOG (*17*). Functional enrichment analysis was performed with the stats package (https://github.com/SurajGupta/r-source/tree/master/src/library/stats/R) in R to calculate odds ratios and Fisher exact tests with p-values corrected for multiple testing by the Bonferroni adjustment. A p value less than 0.05 and an odds ratio higher than 1.5 were considered statistically significant.

List of ECC Genomes Included in this Study

The Genbank accession numbers (hsp60 cluster, phylogenomic group): AFHR01 (E,VII), AMGJ01 (B,VIII), ANIA01 (B,VIII), ANIC01 (B,VIII), ANID01 (B,VIII), AXLJ01 (D,III), AXLK01 (A,VI), AYIE01 (B,VIII), AYIG01 (D,III), AYIK01 (B,VIII), AYIM01 (D,III), AYIR01 (A,VI), AYIY01 (A,VI), AYJE01 (D,III), CP008823 (B,VIII), CP008897 (D,III), CP008905 (D,III), CP009854 (D,III), CP010377 (E,VII), CP010384 (A,VI), CP011572 (B,VIII), CP011581 (B,VIII), CP011584 (B,VIII), CP012165 (A,VI), CP012167 (B,VIII), CP017179 (B,VIII), CP017180 (C,VI), CP017183 (A,VI), CP017186 (D,III), FKHB00 (D,III), FKHE00 (D,III), FKHL00 (D,III), FKHW00 (D,III), FP929040 (B,VIII), JCKK01 (D,III), JCKR01 (A,VI), JCKS01 (A,VI), JCKT01 (B,VIII), JCKX01 (B,VIII), JCLC01 (B,VIII), JCLD01 (B,VIII), JCLF01 (A,VI), JCLG01 (D,III), JCLH01 (A,VI), JCLJ01 (B,VIII), JCLK01 (B,VIII), JCLL01 (D,III), JCLM01 (D,III), JCLN01 (D,III), JCLO01 (B,VIII), JCLP01 (D,III), JCLQ01 (A,VI), JCLR01 (B,VIII), JCLS01 (D,III), JCLT01 (D,III), JCLU01 (A,VI), JCLV01 (D,III), JCLW01 (B,VIII), JJNL01 (D,III), JMUP01 (A,VI), JMUT01 (B,VIII), JQGO01 (B,VIII), JRFQ01 (B,VIII), JSBO01 (A,VI), JTBZ02 (A,VI), JTCA02 (A,VI), JTCB02 (A,VI), JTCC02 (A,VI), JTCD02 (A,VI), JTCE02 (A,VI), JTCF02 (A,VI), JTCG02 (A,VI), JTCH02 (A,VI), JTEP01 (A,VI), JUHV01 (A,VI), JUHW01 (A,VI), JUHX01 (A,VI), JUHY01 (B,VIII), JUIA01 (A,VI), JUIB01 (A,VI), JUIC01 (A,VI), JUID01 (A,VI), JUIE01 (A,VI), JULZ01 (B,VIII), JUNO01 (C,VI), JUXX01 (B,VIII), JVBS01 (B,VIII), JVGX01 (B,VIII), JVKL01 (B,VIII), JVNW01 (D,III), JVOV01 (B,VIII), JVYD01 (B,VIII), JVZA01 (C,VI), JVZC01 (B,VIII), JWBJ01 (A,VI), JWRO01 (A,VI), JWRS01 (A,VI), JYGA02 (A,VI), JYLQ01 (A,VI), JYLR01 (A,VI), JYLU01 (A,VI), JYLW01 (A,VI), JYLY01 (A,VI), JYLZ01 (A,VI), JYMA01 (A,VI),

JYMB01 (A,VI), JYMD01 (A,VI), JYMG01 (B,VIII), JYMI01 (A,VI), JYMJ01 (A,VI), JYMK01 (A,VI), JYML01 (A,VI), JYMN01 (A,VI), JYMO01 (A,VI), JYMP01 (A,VI), JYMQ01 (A,VI), JZCV01 (A,VI), JZCW01 (A,VI), JZCY01 (A,VI), JZDA01 (D,III), JZDB01 (A,VI), JZDC01 (A,VI), JZDD01 (A,VI), JZDE01 (A,VI), JZDG01 (A,VI), JZKC01 (A,VI), JZXQ01 (A,VI), JZXS01 (B,VIII), JZXT01 (A,VI), JZXU01 (B,VIII), JZXV01 (A,VI), JZXW01 (B,VIII), JZXX01 (B,VIII), JZYB01 (A,VI), JZYD01 (A,VI), JZYE01 (A,VI), JZYF01 (A,VI), JZYK01 (A,VI), JZYM01 (B,VIII), JZYN01 (A,VI), JZYO01 (A,VI), JZYP01 (B,VIII), JZYQ01 (D,III), JZYT01 (B,VIII), JZYU01 (A,VI), JZYW01 (B,VIII), JZYY01 (D,III), JZYZ01 (B,VIII), JZZA01 (A,VI), JZZC01 (E,VII), JZZD01 (B,VIII), JZZE01 (B,VIII), JZZH01 (B,VIII), JZZK01 (A,VI), JZZL01 (A,VI), JZZM01 (A,VI), JZZN01 (D,III), JZZO01 (D,III), JZZP01 (E,VII), JZZQ01 (D,III), JZZR01 (A,VI), JZZS01 (A,VI), JZZT01 (D,III), JZZU01 (B,VIII), JZZV01 (A,VI), KI535567 (A,VI), KQ089967 (A,VI), KQ759758 (B,VIII), LAAD01 (B,VIII), LAAE01 (B,VIII), LAAF01 (A,VI), LAAG01 (A,VI), LAAH01 (A,VI), LAAI01 (A,VI), LAAJ01 (A,VI), LAAK01 (A,VI), LAAL01 (A,VI), LAAM01 (A,VI), LAAN01 (B,VIII), LAAQ01 (A,VI), LAAR01 (A,VI), LAAS01 (A,VI), LAAT01 (A,VI), LAAV01 (A,VI), LAAW01 (A,VI), LAAY01 (B,VIII), LAAZ01 (A,VI), LABA01 (A,VI), LABB01 (B,VIII), LABC01 (A,VI), LBLX01 (D,III), LBMV01 (A,VI), LDCB01 (B,VIII), LDCC01 (C,VI), LDCD01 (B,VIII), LDCG01 (A,VI), LEDB01 (B,VIII), LEDD01 (B,VIII), LEDE01 (B,VIII), LEDF01 (D,III), LEDG01 (B,VIII), LEDJ01 (A,VI), LEDK01 (A,VI), LEDO01 (B,VIII), LEDP01 (B,VIII), LEDS01 (B,VIII), LEDU01 (B,VIII), LEDV01 (B,VIII), LEDY01 (B,VIII), LEDZ01 (B,VIII), LEEA01 (B,VIII), LEEB01 (B,VIII), LEED01 (D,III), LEEE01 (B,VIII), LEEF01 (B,VIII), LEEG01 (B,VIII), LEEH01 (B,VIII), LEEI01 (E,VII), LEEM01 (B,VIII), LEEN01 (B,VIII), LETB01 (A,VI), LETC01 (A,VI), LETD01 (B,VIII), LETE01 (D,III), LETF01 (B,VIII), LETG01 (A,VI), LETH01 (B,VIII), LETI01 (D,III), LETJ01 (A,VI), LETK01 (B,VIII), LETL01 (B,VIII), LETM01 (A,VI), LETN01 (B,VIII), LETO01 (A,VI), LETP01 (B,VIII), LFHB01 (A,VI), MKEQ01 (E,VII), AEXB01 (F,na), AGSY00 (H,XII), ALNS01 (M,IV), ATCK01 (I,V), ATHX01 (M,IV), AYID01 (M,IV), AYIP01 (J,I), AYJA01 (M,IV), AYJF01 (Q,II), AYJH01 (Q,II), AYJI01 (Q,II), AYJO01 (M,IV), AZUA01 (J,I), AZUB01 (K,na), AZXO01 (R,IX), AZXZ01 (K,na), CP006580 (I,V), CP009756 (G,XI), CP009850 (Q,II), CP010512 (J,I), CP011591 (J,I), CP012162 (M,IV), CP014993 (J,I), CP016906 (G,XI), CP017181 (Q,II), CP017184 (M,IV), CP017279 (I,V), FKLS00 (Q,II),

JACW01 (K,na), JALR01 (N,na), JALW01 (G,XI), JCKL01 (J,I), JCKQ01 (M,IV), JCKW01 (Q,II), JCLA01 (I,V), JCLB01 (R,IX), JCLE01 (I,V), JCLI01 (Q,II), JDWG01 (P,na), JDWH01 (P,na), JFHW01 (M,IV), JMUQ01 (Q,II), JMUS01 (M,IV), JMUU01 (M,IV), JMUV01 (Q,II), JSWY01 (H,XII), JSZC01 (M,IV), JTBF01 (J,I), JTLO01 (I,V), JUKN01 (J,I), JUMS01 (J,I), JUOY01 (J,I), JUQP01 (Q,II), JUTR01 (Q,II), JUXG01 (J,I), JUZJ01 (P,na), JUZK01 (Q,II), JUZL01 (R,IX), JUZN01 (R,IX), JUZQ01 (P,na), JVAE01 (Q,II), JVAG01 (I,V), JVBX01 (G,XI), JVBY01 (G,XI), JVCE01 (G,XI), JVFX01 (Q,II), JVIB01 (L,na), JVIL01 (L,na), JVLF01 (J,I), JVMT01 (M,IV), JVND01 (M,IV), JVPP01 (R,IX), JVQI01 (R,IX), JVQL01 (R,IX), JVQZ01 (R,IX), JVRK01 (R,IX), JVSD01 (R,IX), JVTR01 (Q,II), JVWV01 (M,IV), JWAA01 (J,I), JWAF01 (M,IV), JWAU01 (K,na), JWAV01 (K,na), JWBX01 (J,I), JWCB01 (K,na), JWCF01 (J,I), JWCN01 (J,I), JWET01 (Q,II), JWFJ01 (Q,II), JWFR01 (J,I), JWGJ01 (J,I), JWGM01 (J,I), JWPV01 (M,IV), JWPX01 (G,XI), JXAE01 (G,XI), JYME01 (Q,II), JYMF01 (J,I), JYMH01 (M,IV), JYMM01 (J,I), JZCX01 (J,I), JZDF01 (R,IX), JZXR01 (Q,II), JZXZ01 (L,na), JZYA01 (L,na), JZYC01 (M,IV), JZYG01 (G,XI), JZYH01 (Q,II), JZYJ01 (M,IV), JZYR01 (M,IV), JZYS01 (Q,II), JZYX01 (N,na), JZZB01 (R,IX), JZZF01 (M,IV), JZZG01 (J,I), JZZI01 (M,IV), JZZX01 (M,IV), LAAP01 (J,I), LABD01 (M,IV), LDCE01 (J,I), LDCH01 (J,I), LDCI01 (G,XI), LDCJ01 (Q,II), LDCK01 (M,IV), LDCL01 (Q,II), LECX01 (J,I), LECY01 (Q,II), LECZ01 (O,na), LEDA01 (J,I), LEDC01 (Q,II), LEDH01 (J,I), LEDI01 (M,IV), LEDL01 (G,XI), LEDN01 (L,na), LEDQ01 (R,IX), LEDR01 (H,XII), LEDT01 (L,na), LEDW01 (Q,II), LEDX01 (I,V), LEEC01 (Q,II), LEEJ01 (R,IX), LEEK01 (Q,II), LEEL01 (Q,II), LEEO01 (I,V), LEEP01 (M,IV), LEEQ01 (Q,II), LEER01 (I,V), LEES01 (M,IV), LEET01 (M,IV), LETR01 (N,na), LFLG01 (Q,II), LFLH01 (H,XII), LGIV01 (I,V), LT160614 (G,XI), LVTZ00 (H,XII), LVUS00 (H,XII), LVUX01 (Q,II), NC_014121 (G, XI), NC_015968 (na, XIII), NC_016514 (I, V), NC_018079 (H, XII), NC_018405 (Q,II). (na, not attributed).

Biofilm Formation and Epithelial Cell Adhesion Assays

The initiation of biofilm formation was assayed by the ability of cells to adhere to the wells of 96-well microtiter dishes made of polyvinylchloride plastic as previously described (*18*). Biofilm formation was detected after 3h of incubation at room temperature by determining the extent of crystal violet-stained cells attached to a surface at 595 nm. The commensal *E. coli* K-12 strain TG1 carrying a F-conjugative plasmid that promotes biofilm formation (*19*) was used as a positive control strain for biofilm formation.

HT-29 intestinal epithelial cells were purchased from ATCC and maintained in an atmosphere containing 5% CO_2 at 37°C in Dulbecco's modified Eagle's medium. They were seeded at a density of 2×10^5 cells/cm^2 in culture plates (Falcon) for 48 H. Cells were infected at a multiplicity of infection of 10 bacteria per cell. Infected cells were centrifuged at 900 g for 10 min at 25°C, maintained at 37°C for 3 hours and then washed three times in phosphate-buffered saline (PBS; pH 7.2). The epithelial cells were then lysed with 1% Triton X-100 (Sigma) in deionized water. Samples were diluted and plated onto Luria-Bertani (LB) agar plates to determine the number of CFU corresponding to the total number of cell-associated bacteria. The adherent-invasive *E. coli* strain LF82 (*20*) was used as positive control. The adhesion index was expressed as the mean number of associated bacteria per epithelial cell.

The data were compared by the Kruskal-Wallis test with Dunn's post hoc tests. The p-values <0.05 were rated as significant.

Results

Clinical Data

The index case (patient 1, P1) was a 67-year-old woman hospitalized for a kidney transplant. Following surgery, a CPE designated C45 was isolated from a urine sample. However, P1 did not develop an infection and was only colonized. Five months after she was discharged, a second CPE strain designated C46 was isolated from the urine of a 72-year-old man (patient 2, P2) admitted to the intensive care unit (ICU) for endarterectomy and tracheotomy. During his 106 days of hospitalization, the patient developed septic shock. Patient 3 (P3) was a 52-year-old woman admitted to the ICU for an undetermined recurrent septic shock following bowel resection and laparotomy. Two weeks after the isolation of strain C46 from P2, a CPE strain designated C47 was identified from the urine of P3. Patient 4 (P4) was a 69-year-old man hospitalized for a kidney transplant. Two weeks after the isolation of C47 from P3, a CPE strain designated C48 was isolated from the urine of P4. Patient 5 (P5) was an 87-year-old man admitted to the medical unit and then to the ICU for a consciousness disorder. A CPE strain designated C308 was isolated from a urine sample of P5 3 months after the isolation of strain C48. Two months after the isolation of C308, CPE strain C310 was isolated from a skin sample taken from an 84-year-old woman admitted to the medical unit for the treatment of a necrotic

ulcer (patient 6, P6). Patient 7 (P7) was an 82-year-old man admitted to the surgical unit to undergo lithotrity. One month after the isolation of C310, CPE strain C309 was isolated from the operative peritoneal fluid of P7.

References

1. Kluytmans-van den Bergh MFQ, Rossen JWA, Bruijning-Verhagen PCJ, Bonten MJM, Friedrich AW, Vandenbroucke-Grauls CMJE, et al. Whole-genome multilocus sequence typing of extended-spectrum-Beta-lactamase-producing *Enterobacteriaceae*. J Clin Microbiol. 2016;54:2919–27. PubMed http://dx.doi.org/10.1128/JCM.01648-16

2. Van der Auwera GA, Carneiro MO, Hartl C, Poplin R, Del Angel G, Levy-Moonshine A, et al. From FastQ data to high confidence variant calls: the Genome Analysis Toolkit best practices pipeline. Curr Protoc Bioinforma. 2013;43:11.10.1–33. PubMed https://doi.org/10.1002/0471250953.bi1110s43

3. Koboldt DC, Chen K, Wylie T, Larson DE, McLellan MD, Mardis ER, et al. VarScan: variant detection in massively parallel sequencing of individual and pooled samples. Bioinformatics. 2009;25:2283–5. PubMed http://dx.doi.org/10.1093/bioinformatics/btp373

4. Kaas RS, Leekitcharoenphon P, Aarestrup FM, Lund O. Solving the problem of comparing whole bacterial genomes across different sequencing platforms. PLoS One. 2014;9:e104984. PubMed http://dx.doi.org/10.1371/journal.pone.0104984

5. Stamatakis A. RAxML version 8: a tool for phylogenetic analysis and post-analysis of large phylogenies. Bioinformatics. 2014;30:1312–3. PubMed http://dx.doi.org/10.1093/bioinformatics/btu033

6. Hunt M, Mather AE, Sánchez-Busó L, Page AJ, Parkhill J, Keane JA, et al. ARIBA: rapid antimicrobial resistance genotyping directly from sequencing reads. Microb Genom. 2017;3:e000131. PubMed http://dx.doi.org/10.1099/mgen.0.000131

7. Zankari E, Hasman H, Cosentino S, Vestergaard M, Rasmussen S, Lund O, et al. Identification of acquired antimicrobial resistance genes. J Antimicrob Chemother. 2012;67:2640–4. PubMed http://dx.doi.org/10.1093/jac/dks261

8. Jia B, Raphenya AR, Alcock B, Waglechner N, Guo P, Tsang KK, et al. CARD 2017: expansion and model-centric curation of the comprehensive antibiotic resistance database. Nucleic Acids Res. 2017;45(D1):D566–73. PubMed http://dx.doi.org/10.1093/nar/gkw1004

9. Slater GSC, Birney E. Automated generation of heuristics for biological sequence comparison. BMC Bioinformatics. 2005;6:31. PubMed http://dx.doi.org/10.1186/1471-2105-6-31

10. Treangen TJ, Ondov BD, Koren S, Phillippy AM. The Harvest suite for rapid core-genome alignment and visualization of thousands of intraspecific microbial genomes. Genome Biol. 2014;15:524. PubMed http://dx.doi.org/10.1186/s13059-014-0524-x

11. Price MN, Dehal PS, Arkin AP. FastTree 2—approximately maximum-likelihood trees for large alignments. PLoS One. 2010;5:e9490. PubMed http://dx.doi.org/10.1371/journal.pone.0009490

12. Hoffmann H, Roggenkamp A. Population genetics of the nomenspecies *Enterobacter cloacae*. Appl Environ Microbiol. 2003;69:5306–18. PubMed http://dx.doi.org/10.1128/AEM.69.9.5306-5318.2003

13. Richter M, Rosselló-Móra R. Shifting the genomic gold standard for the prokaryotic species definition. Proc Natl Acad Sci U S A. 2009;106:19126–31. PubMed http://dx.doi.org/10.1073/pnas.0906412106

14. Seemann T. Prokka: rapid prokaryotic genome annotation. Bioinformatics. 2014;30:2068–9. PubMed http://dx.doi.org/10.1093/bioinformatics/btu153

15. Page AJ, Cummins CA, Hunt M, Wong VK, Reuter S, Holden MTG, et al. Roary: rapid large-scale prokaryote pan genome analysis. Bioinformatics. 2015;31:3691–3. PubMed http://dx.doi.org/10.1093/bioinformatics/btv421

16. Tatusov RL, Fedorova ND, Jackson JD, Jacobs AR, Kiryutin B, Koonin EV, et al. The COG database: an updated version includes eukaryotes. BMC Bioinformatics. 2003;4:41. PubMed http://dx.doi.org/10.1186/1471-2105-4-41

17. Jensen LJ, Julien P, Kuhn M, von Mering C, Muller J, Doerks T, et al. eggNOG: automated construction and annotation of orthologous groups of genes. Nucleic Acids Res. 2007;36(Database):D250–4. PubMed http://dx.doi.org/10.1093/nar/gkm796

18. O'Toole GA, Kolter R. Initiation of biofilm formation in *Pseudomonas fluorescens* WCS365 proceeds via multiple, convergent signalling pathways: a genetic analysis. Mol Microbiol. 1998;28:449–61. PubMed http://dx.doi.org/10.1046/j.1365-2958.1998.00797.x

19. Latimer J, Stokes SL, Graham AI, Bunch J, Jackson RJ, McLeod CW, et al. A novel method for exploring elemental composition of microbial communities: laser ablation-inductively coupled plasma-mass spectrometry of intact bacterial colonies. J Microbiol Methods. 2009;79:329–35. PubMed http://dx.doi.org/10.1016/j.mimet.2009.10.001

20. Boudeau J, Barnich N, Darfeuille-Michaud A. Type 1 pili-mediated adherence of *Escherichia coli* strain LF82 isolated from Crohn’s disease is involved in bacterial invasion of intestinal epithelial cells. Mol Microbiol. 2001;39:1272–84. PubMed http://dx.doi.org/10.1111/j.1365-2958.2001.02315.x

21. Hoffmann H, Stindl S, Ludwig W, Stumpf A, Mehlen A, Monget D, et al. *Enterobacter hormaechei* subsp. oharae subsp. nov., *E. hormaechei* subsp. *hormaechei* comb. nov., and E. hormaechei subsp. steigerwaltii subsp. nov., three new subspecies of clinical importance. J Clin Microbiol. 2005;43:3297–303. PubMed http://dx.doi.org/10.1128/JCM.43.7.3297-3303.2005

22. Delcher AL, Salzberg SL, Phillippy AM. Using MUMmer to identify similar regions in large sequence sets. Curr Protoc Bioinformatics. 2003; Chapter 10:Unit 10.3 PubMed http://dx.doi.org/10.1002/0471250953.bi1003s00

Technical Appendix Table 1. Fermentation tests of *Enterobacter cloacae* complex isolates performed by using API 50CH

Isolates	3-hydroxy-butyrate	Amidon	Amygdaline	Arbutine	citrate	D-Adonitol	D-Arabinose	D-Arabitol	D-Celliobiose	D-Fucose	D-Fructose	D-Galactose	D-Glucose	D-Lactate	D-Lyxose	D-Maltose	D-Mannose	D-Mannitol	D-Melezitose	D-Melibiose	D-Raffinose	D-Ribose	D-Saccharose	D-Sorbitol	D-Tagaose	D-Trehalose
ST-873 (n = 8)	-	-	+	+	+	-	-	-	+	-	+	+	+	+	-	+	+	+	-	+	+	+	+	-	-	+
ST-118 (n = 2)	-	-	+	+	+	-	+	-	+	-	+	+	+	+	-	+	+	+	-	+	-	+	+	+	-	+
ST-110 (n = 1)	-	-	+	+	+	+	+	+	+	-	+	+	+	+	-	+	+	+	-	+	+	+	+	+	-	+

Isolates	D-Turanose	D-Xylose	Dulcitol	Erythritol	Esculin / Ferric citrate	Gentibiose	Glycerol	Glycogene	Inositol	Inuline	L-Arabinose	L-Arabitol	L-Fucose	L-Rahmnose	L-Sorbose	L-Xylose	Methyl-αD-Glycopyranoside	Methyl-αD-Mannopyranoside	Methyl-βD-Xylopyranoside	N-Acetyl-Glucosamine	ONPG	Potassium 2-CetoGluconate	Potassium 5-CetoGluconate	Potassium gluconate	Salicine	Xylitol
ST-873 (n = 8)	-	+	-	-	-	+	+	-	-	-	+	-	-	+	-	-	-	-	-	+	+	+	-	+	+	-
ST-118 (n = 2)	-	+	-	-	+	+	+	-	-	-	+	-	+	+	-	-	+	-	-	+	+	+	-	+	+	-
ST-110 (n = 1)	+	+	-	-	-	+	+	-	-	-	+	-	+	+	-	-	+	-	-	+	+	+	-	+	-	-

Technical Appendix Table 2. Biochemical differentiation of the isolates ST-873 among *Enterobacter hormaechei* subspecies and other relevant species of the *E. cloacae complex**

	D-Adonitol	D-Arabitol	D-Sorbitol	D-Melibiose	L-Fucose	Esculin	Dulcitol
E. cloacae complex ST-873 (n = 8) (*21*)	–	–	–	+	–	–	–
E. hormaechei subsp. *oharae*	–	–	+	+	V	V	–
E. hormaechei subsp. *hormaechei*	–	–	–	–	+	–	+
E. hormaechei subsp. *steigerwaltii*	+	+	+	+	+	–	–
E. asburiae	–	–	+	+	–	+	–
E. kobei	–	–	+	+	–	–	V
E. cloacae	–	–	+	+	–	–	–
E. dissolvens	V	–	+	+	V	+	–

* –, absence of fermentation; +, fermentation; V, variable among isolates.

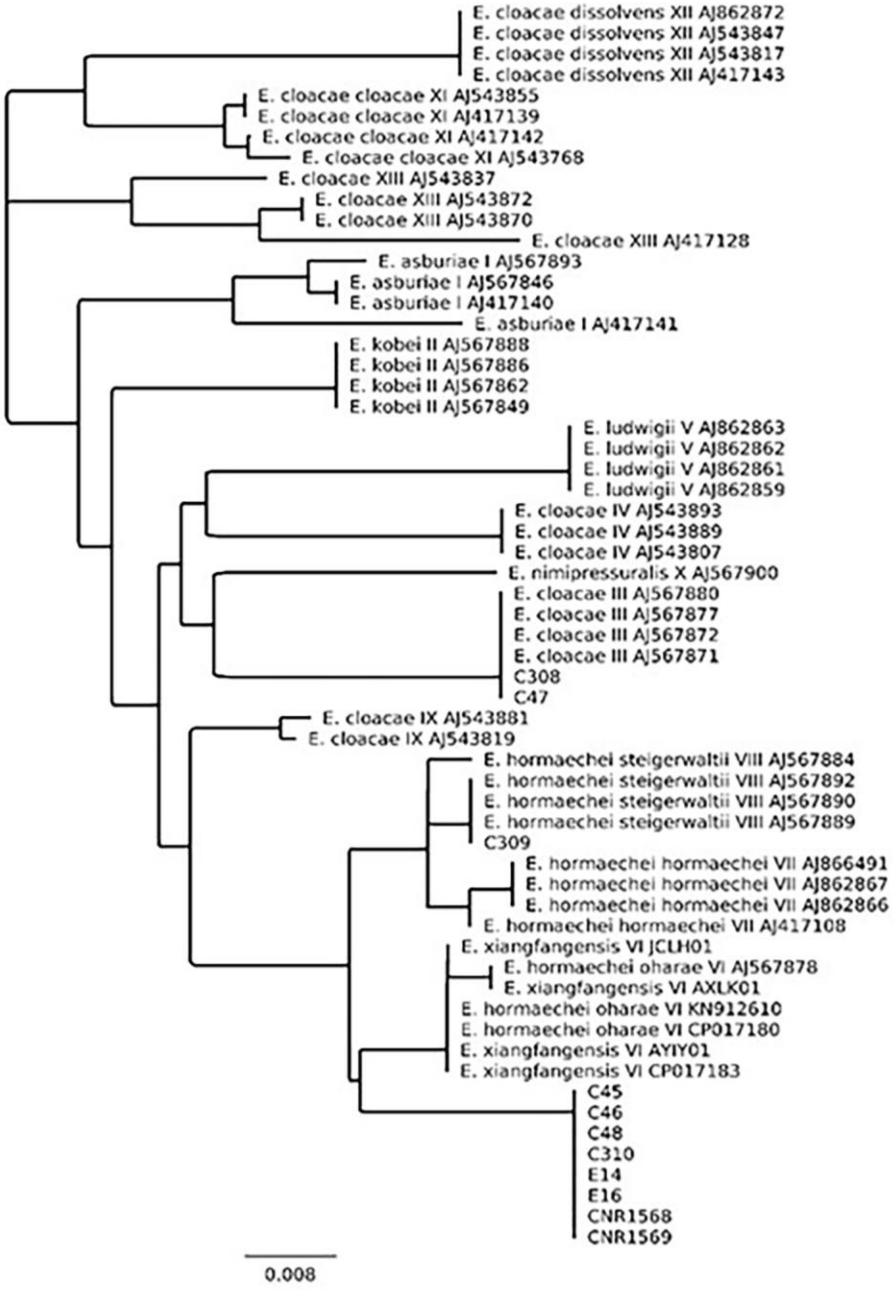

Technical Appendix Figure 1: Neighbor-joining tree based on the *hsp60* gene of representative *Enterobacter cloacae* complex strains including type and reference strains. The isolates C45, C46, C48, C310, E14, E16, CNR1568 and CNR1569 form a new *hsp60* genetic cluster. The scale gives the Jukes-Cantor distance along the branches. The leaves are labeled by species name, *hsp60* genetic group and the corresponding GenBank accession number.

Meta-cluster	Name	hsp60 cluster	Genomic cluster	A	B	C	D	E	S	F	G	H	I	J	K	L	M	N	O	P	Q	R	NA
hormaechei	*E. xiangfangensis*	VI	A (n=110)	0.91																			
	E. hormaechei subsp. *steigerwaltii*	VIII	B (n=83)	0.84	0.89																		
	E. hormaechei subsp. *oharae*	VI	C (n=4)	0.87	0.87	0.94																	
	E. hormaechei	III	D (n=37)	0.84	0.83	0.83	0.89																
	E. hormaechei subsp. *hormaechei*	VII	E (n=6)	0.82	0.82	0.82	0.81	0.89															
	ST-873	NA	S (n=8)	0.81	0.80	0.81	0.80	0.76	0.98														
cloacae	*E. mori*	NA	F (n=1)	0.76	0.76	0.75	0.75	0.72	0.70	1.00													
	E. cloacae subsp. *cloacae*	XI	G (n=13)	0.77	0.77	0.76	0.76	0.73	0.72	0.78	0.89												
	E. cloacae subsp. *dissolvens*	XII	H (n=7)	0.76	0.76	0.75	0.75	0.72	0.71	0.80	0.86	0.93											
	E. ludwigii	NA	I (n=12)	0.77	0.78	0.77	0.77	0.73	0.72	0.79	0.81	0.82	0.92										
	E. asburiae	I	J (n=29)	0.77	0.76	0.77	0.76	0.73	0.72	0.78	0.79	0.81	0.80	0.88									
	E. cloacae complex	NA	K (n=6)	0.75	0.75	0.75	0.74	0.71	0.70	0.78	0.79	0.81	0.80	0.82	0.93								
	E. cloacae complex	NA	L (n=6)	0.75	0.75	0.75	0.74	0.71	0.71	0.73	0.76	0.77	0.77	0.79	0.79	0.94							
	E. cloacae complex	NA	M (n=30)	0.75	0.75	0.75	0.74	0.71	0.71	0.75	0.77	0.78	0.77	0.80	0.81	0.80	0.87						
	E. cloacae complex	NA	N (n=3)	0.73	0.72	0.72	0.71	0.69	0.68	0.74	0.76	0.78	0.75	0.76	0.79	0.76	0.78	0.89					
	E. cloacae complex	NA	O (n=1)	0.74	0.74	0.73	0.73	0.70	0.69	0.78	0.79	0.81	0.78	0.78	0.80	0.77	0.78	0.78	1.00				
	E. cloacae complex	NA	P (n=4)	0.74	0.74	0.74	0.74	0.71	0.69	0.75	0.77	0.78	0.78	0.80	0.82	0.80	0.80	0.79	0.80	0.94			
	E. kobei	II	Q (n=34)	0.77	0.77	0.77	0.76	0.74	0.73	0.76	0.79	0.79	0.80	0.79	0.79	0.80	0.81	0.78	0.80	0.76	0.89		
	E. cloacae complex	IX	R (n=14)	0.80	0.80	0.79	0.79	0.75	0.75	0.79	0.82	0.82	0.82	0.81	0.82	0.82	0.83	0.80	0.83	0.77	0.83	0.91	
	E. cloacae complex	XIII	NA (n=1)	0.73	0.75	0.73	0.73	0.70	0.69	0.75	0.76	0.76	0.77	0.75	0.77	0.76	0.76	0.74	0.78	0.76	0.75	0.76	1.00

Technical Appendix Figure 2: Percentages of conserved DNA (PCD) calculated from Blast software (http://blast.ncbi.nlm.nih.gov/Blast.cgi) pairwise comparisons of ST-873 genomes and 401 representative genomes of *E. cloacae complex*. (NA, non-attributed)

Meta-cluster	Name	Hsp60 cluster	Genomic cluster	A	B	C	D	E	S	F	G	H	I	J	K	L	M	N	O	P	Q	R	NA
hormaechei	*E. xiangfangensis*	VI	A (n=110)	0.93																			
	E. hormaechei subsp. *steigerwaltii*	VIII	B (n=83)	0.86	0.91																		
	E. hormaechei subsp. *oharae*	VI	C (n=4)	0.86	0.87	0.95																	
	E. hormaechei	III	D (n=37)	0.85	0.86	0.88	0.92																
	E. hormaechei subsp. *hormaechei*	VII	E (n=6)	0.80	0.81	0.83	0.81	0.91															
	ST-873	NA	S (n=8)	0.84	0.84	0.87	0.84	0.82	1.00														
cloacae	E. mori	NA	F (n=1)	0.75	0.76	0.77	0.75	0.73	0.71	1.00													
	E. cloacae subsp. *cloacae*	XI	G (n=13)	0.76	0.76	0.78	0.76	0.75	0.72	0.79	0.91												
	E. cloacae subsp. *dissolvens*	XII	H (n=7)	0.75	0.76	0.77	0.75	0.74	0.71	0.81	0.88	0.95											
	E. ludwigii	NA	I (n=12)	0.74	0.75	0.76	0.75	0.73	0.70	0.77	0.79	0.80	0.93										
	E. asburiae	I	J (n=29)	0.74	0.75	0.77	0.75	0.73	0.71	0.77	0.77	0.79	0.79	0.90									
	E. cloacae complex	NA	K (n=6)	0.73	0.74	0.75	0.73	0.72	0.70	0.78	0.77	0.79	0.80	0.85	0.94								
	E. cloacae complex	NA	L (n=6)	0.75	0.75	0.78	0.75	0.75	0.72	0.75	0.77	0.78	0.79	0.85	0.83	0.95							
	E. cloacae complex	NA	M (n=30)	0.75	0.75	0.78	0.75	0.75	0.72	0.77	0.78	0.79	0.80	0.86	0.85	0.83	0.89						
	E. cloacae complex	NA	N (n=3)	0.73	0.73	0.75	0.73	0.73	0.69	0.77	0.79	0.80	0.78	0.83	0.83	0.79	0.81	0.91					
	E. cloacae complex	NA	O (n=1)	0.75	0.76	0.77	0.75	0.75	0.72	0.82	0.83	0.85	0.82	0.85	0.86	0.81	0.82	0.81	1.00				
	E. cloacae complex	NA	P (n=4)	0.70	0.71	0.72	0.70	0.69	0.66	0.73	0.74	0.75	0.76	0.81	0.82	0.79	0.78	0.77	0.76	0.95			
	E. kobei	II	Q (n=34)	0.76	0.76	0.78	0.76	0.76	0.73	0.76	0.78	0.78	0.80	0.83	0.82	0.81	0.81	0.78	0.78	0.80	0.91		
	E. cloacae complex	IX	R (n=14)	0.76	0.77	0.78	0.77	0.75	0.73	0.78	0.79	0.80	0.81	0.83	0.83	0.81	0.82	0.78	0.79	0.80	0.83	0.92	
	E. cloacae complex	XIII	NA (n=1)	0.69	0.71	0.71	0.70	0.68	0.65	0.71	0.71	0.72	0.73	0.73	0.73	0.70	0.71	0.68	0.71	0.73	0.71	0.73	1.00

Technical Appendix Figure 3: Percentages of conserved DNA (PCD) calculated from MUMer (*22*) pairwise comparisons of ST-873 genomes and 401 genomes of *E. cloacae complex*. (NA, non-attributed)

Meta-cluster	Name	hsp60 cluster	Genomic cluster	A	B	C	D	E	S	F	G	H	I	J	K	L	M	N	O	P	Q	R	NA
hormaechei	*E. xiangfangensis*	VI	A (n=110)	0.99																			
	E. hormaechei subsp. *steigerwaltii*	VIII	B (n=83)	0.97	0.99																		
	E. hormaechei subsp. *oharae*	VI	C (n=4)	0.97	0.98	1.00																	
	E. hormaechei	III	D (n=37)	0.96	0.96	0.96	0.99																
	E. hormaechei subsp. *hormaechei*	VII	E (n=6)	0.95	0.95	0.95	0.95	1.00															
	ST-873	NA	S (n=8)	0.94	0.94	0.94	0.94	0.93	1.00														
cloacae	E. mori	NA	F (n=1)	0.88	0.89	0.89	0.88	0.89	0.89	1.00													
	E. cloacae subsp. *cloacae*	XI	G (n=13)	0.88	0.88	0.88	0.88	0.88	0.88	0.88	0.99												
	E. cloacae subsp. *dissolvens*	XII	H (n=7)	0.88	0.88	0.88	0.88	0.88	0.88	0.88	0.95	0.99											
	E. ludwigii	NA	I (n=12)	0.87	0.87	0.87	0.87	0.87	0.87	0.88	0.88	0.88	0.99										
	E. asburiae	I	J (n=29)	0.89	0.89	0.89	0.89	0.89	0.89	0.90	0.89	0.89	0.89	0.98									
	E. cloacae complex	NA	K (n=6)	0.88	0.88	0.89	0.88	0.88	0.88	0.90	0.89	0.89	0.89	0.95	0.99								
	E. cloacae complex	NA	L (n=6)	0.89	0.89	0.89	0.88	0.89	0.89	0.90	0.89	0.89	0.88	0.93	0.93	1.00							
	E. cloacae complex	NA	M (n=30)	0.89	0.89	0.89	0.89	0.89	0.89	0.90	0.89	0.89	0.89	0.93	0.94	0.93	0.98						
	E. cloacae complex	NA	N (n=3)	0.88	0.88	0.88	0.88	0.88	0.88	0.89	0.89	0.89	0.89	0.92	0.92	0.91	0.92	0.99					
	E. cloacae complex	NA	O (n=1)	0.88	0.88	0.88	0.88	0.88	0.88	0.90	0.89	0.89	0.89	0.93	0.93	0.92	0.92	0.93	1.00				
	E. cloacae complex	NA	P (n=4)	0.88	0.88	0.88	0.87	0.88	0.88	0.88	0.88	0.89	0.88	0.91	0.91	0.90	0.90	0.90	0.91	0.99			
	E. kobei	II	Q (n=34)	0.88	0.88	0.88	0.88	0.88	0.88	0.89	0.89	0.89	0.88	0.91	0.91	0.91	0.91	0.90	0.90	0.89	0.99		
	E. cloacae complex	IX	R (n=14)	0.89	0.89	0.89	0.89	0.89	0.90	0.90	0.89	0.89	0.88	0.92	0.91	0.91	0.92	0.90	0.91	0.90	0.91	0.99	
	E. cloacae complex	XIII	NA (n=1)	0.87	0.87	0.87	0.87	0.87	0.87	0.87	0.87	0.87	0.87	0.87	0.87	0.87	0.87	0.87	0.87	0.87	0.87	0.87	1.00

Technical Appendix Figure 4: Average nucleotide identities (ANI) calculated from MUMer (*22*) pairwise comparisons of ST-873 genomes and 401 genomes of *E. cloacae complex*. (NA, non-attributed)

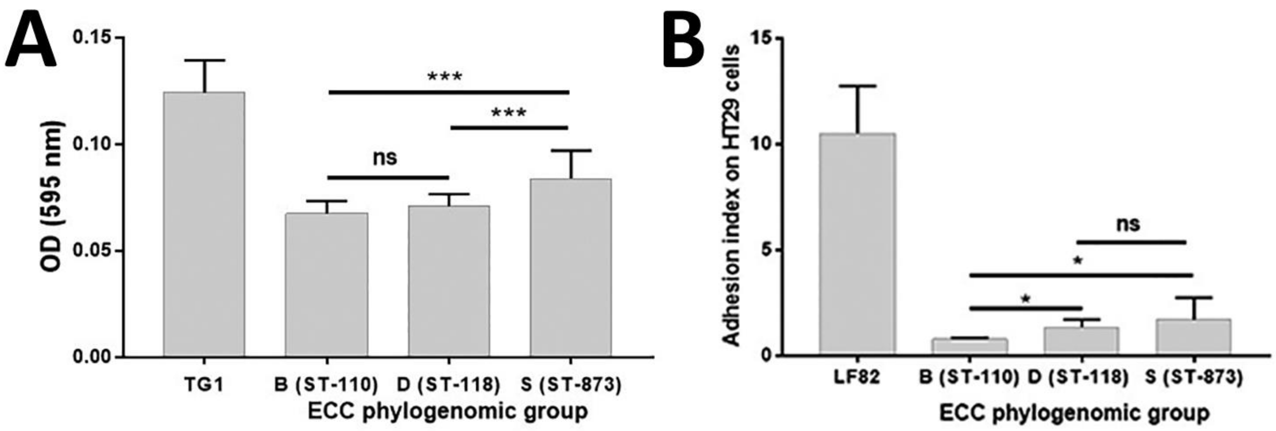

Technical Appendix Figure 5: Ability of A) ECC isolates to initiate biofilm formation on PCV and B) to adhere to HT29 intestinal epithelial cells. *E. coli* K12 TG1 was used as positive control for biofilm formation and the *E. coli* reference strain LF82 for adhesion to intestine epithelial cells (Kruskal-Wallis Test; ns, not significant; *, p<0.001; **, p<0.05).
